# Expression of a triple mutational *des*‐pGlu brazzein in transgenic mouse milk

**DOI:** 10.1002/2211-5463.13411

**Published:** 2022-05-03

**Authors:** Rui Lu, Xiaoming Li, Jian Hu, Yong Zhang, Yancui Wang, Le Jin

**Affiliations:** ^1^ Jiangsu Food and Pharmaceutical Science College Huaian China; ^2^ Jiangsu Agri‐Animal Husbandry Vocational College Taizhou China

**Keywords:** *des*‐pGlu brazzein, expression, mammary gland bioreactor, sweeteners, transgenic mouse

## Abstract

Brazzein has excellent potential for use as a sweetener because of its high level of sweetening potency and stability against extreme temperature and pH. It is extracted from the tropical and difficult—to‐cultivate African plant *Pentadiplandra brazzeana*, which hampers its commercial viability. Here we report the mammary‐specific expression of wildtype or triple mutational (H31R/E36D/E41A) *des*‐pGlu brazzeins in the milk of transgenic mice. Using enzyme‐linked immunoassay (ELISA), western blot, and sweetness intensity testing, we confirmed that the triple mutation made the *des*‐pGlu brazzein molecule 10,000 times sweeter than sucrose in a weight base, even after 10 min of incubation at 100 °C; in addition, the triple mutant was also significantly sweeter than the wildtype *des*‐pGlu brazzein. This study provides new insights for producing brazzein or brazzein‐sweetened milk from animals for use in food and healthcare applications.

## Graphical abstract

The commercial production of brazzein has been limited by geographical and cultivational constraints of the source plant, *Pentadiplandra brazzeana*. Lu *et al*. expressed *des*‐pGlu brazzeins in the milk of transgenic mice. The triple‐mutant form of *des*‐pGlu brazzein was 10,000 times sweeter than sucrose in a weight base even after 10 min at 100°, and also significantly sweeter than the wildtype molecule.

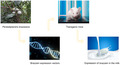

**Abbreviations**
pGlu: pyroglutamic Acid
DIG: digoxin

Excessive sugar consumption can easily lead to obesity [1], diabetes [2], dislypidemia, and coronary heart disease [3], threatening human health. In order to meet the needs for sweet food from people who should reduce their sugar intake, it is urgently needed to develop natural and noncaloric sweeteners with good taste characteristics. An ideal solution is to commercialize sweet‐tasting proteins that do not adversely affect health [4, 5, 6, 7].

Brazzein, one of the eight sweet proteins discovered so far, has attracted the attention of many sweetener researchers. It is a small molecule, which was first discovered in the red berries of the evergreen shrub *Pentadiplandra brazzeana* Baillon [8, 9]. Brazzein, composed of a single chain of 54 amino acid residues (6.5 kDa), has two isoforms in its natural source: the major isoform (~80%), contains a pyroglutamic acid at its N‐terminus, is about 9500 times sweeter than sucrose on a molar basis; the minor isoform (~20%, called *des*‐pGlu‐brazzein), lacks the pyroglutamic acid at its N‐terminus, and is two times sweeter that of the major isoform [8, 10]. In addition to being particularly sweet, brazzein tastes like sugar, and maintains its sweetness at a wide pH range and high temperature [8, 11, 12].

It’s very difficult to obtain brazzein from its natural source [9, 13]; large‐scale production of brazzein will probably require recombinant production in genetically modified bioreactors. Recombinant brazzein has been expressed in a variety of bioreactors, including bacteria [14, 15], yeasts [16, 17, 18], transgenic plants [19] and transgenic mice [13]. Compared with other bioreactors capable of producing recombinant proteins, transgenic animal mammary gland bioreactors have great potential [20, 21, 22]. And it has already been successfully used to express plant proteins, such as thaumatin [23], wildtype brazzein [13] and D12 fatty acid desaturase [24], indicating that plant proteins can be expressed in the milk of transgenic animals.

Many studies on brazzein mutants have shown that the interaction of certain amino acid residues with sweet taste receptors is essential for people to perceive sweet taste [15, 25, 26, 27]. Liu *et al*. reported *des*‐pGlu brazzein expressed in *Escherichia coli* displayed a sweetness threshold of about 1.5 μg·mL^−1^ [10]. Lee *et al*. reported a triple mutation (H31R/E36D/E41A, the 31th histidine into arginine, the 36th glutamic acid into aspartic acid, and the 41th glutamic acid into alanine) of pGlu brazzein in *Escherichia coli*, the sweetness threshold at about 0.889 μg·mL^−1^ [25]. The sweetness change of triple mutational (H31R/E36D/E41A) *des*‐pGlu brazzein is currently unknown.

Here we describe the mammary‐specific expression of triple mutational (H31R/E36D/E41A) *des*‐pGlu and wildtype brazzein in transgenic mice, to explore whether the triple mutant is sweeter than the wildtype, and to evaluate the feasibility of expressing mutational *des*‐pGlu brazzein in transgenic animal milk.

## Materials and methods

### Ethics statement

The study design conformed to the guidelines set by the Declaration of Helsinki, and was approved by the Ethics Committee of Jiangsu Food and Pharmaceutical Science College (Approved ID EC‐20200930). Informed written consent was obtained from all participants.

The study was approved by the Animal Care and Use Committee of Jiangsu Food and Pharmaceutical Science College (Approved ID SKLAB‐20200930). All surgical operations were performed under anesthesia to reduce the suffering of the animals. Mice were housed with a 12/12 h light/dark cycle, and had free access to food and water.

### Construction of the expression vectors

**Fig. 1:**
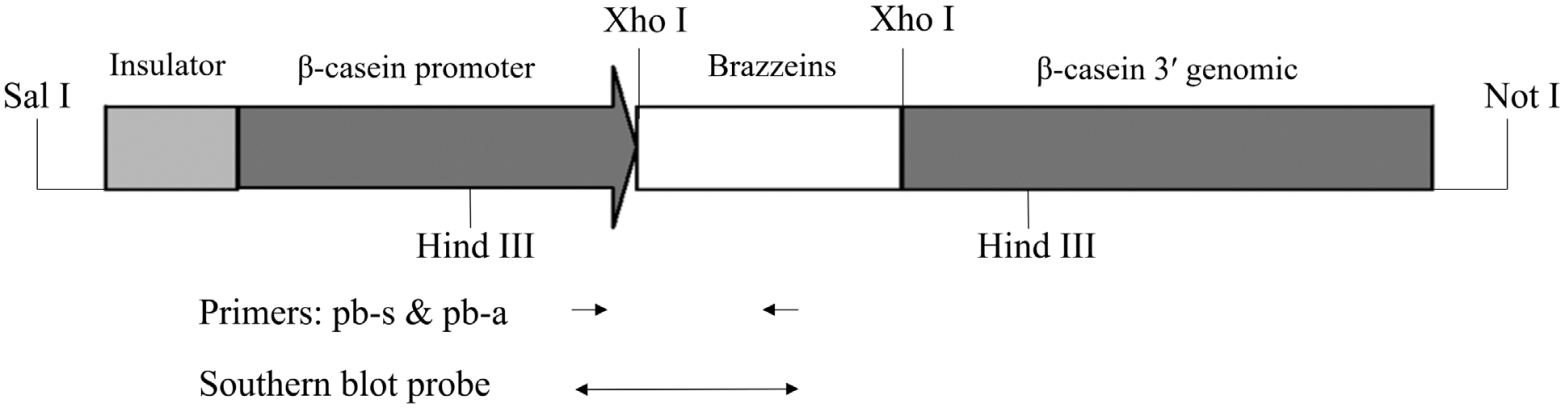
Schematic of pBC1‐brazzein and pBC1‐brazzein‐H31R/E36D/E41A. Insulator: chicken β‐globin insulator (2X); β‐casein promoter: goat beta‐casein promoter. Brazzeins: optimized wildtype or triple mutational (H31R/E36D/E41A) brazzein coding regions; β‐casein 3′ genomic: goat β‐casein downstream genomic fragment; the loci of the primers used for PCR and Southern blot probe production are also marked in the figure.

The wildtype (GenBank accession no. KF013250.1) or triple mutational (H31R/E36D/E41A) *des*‐pGlu brazzein cDNAs were artificially synthesized. The synthesized genes were both chimeras of a kozak sequence, a start codon, a goat beta‐lactoglobulin signaling peptide cDNA, a brazzein mature peptide, and a stop codon. We optimized the codons of the chimeric brazzein cDNAs for better expression in breast epithelial cells of mammals. The synthesized genes were cloned into a commercial pBC1 mammary‐specific expression vector (Invitrogen, Carlsbad, CA, USA) and verified by sequencing. The resulting vectors were designated as pBC1‐brazzein and pBC1‐brazzein‐H31R/E36D/E41A, respectively (Fig. 1).

### Generation of brazzein transgenic mice via microinjection

The expression vectors were double‐digested with *Not* I and *Sal* I, and the eukaryotic part of the expression vector about 19‐kb was purified by the QIAquick gel extraction kit (Qiagen, Germany). The purified DNA fragments were dissolved to 5 ng·µL^−1^ and microinjected into the pronuclear stage wildtype mice zygotes. Subsequently, the microinjected zygotes were transferred into the uterus of pseudopregnant mothers, as described elsewhere [28].

### PCR and Southern blot analysis

Transgenic mice were determined by polymerase chain reaction (PCR) using the primer pairs pb‐s: 5′‐ TCACTGCTCTAATCCCAGAA −3′ and pb‐a: 5′‐ GCGATAGCCAGGGCCACCAG −3′. The theoretical PCR‐amplified product was 592 bp.

Further detection to confirm was done by Southern blotting using the DIG Random Labeling and Detection Kit I (Boster, Wuhan, Hubei, China). Genomic DNA of candidate and wildtype mice and expressions were digested by *Hind* III for 20 h. The PCR‐amplified product was labeled with digoxigenin and used as the probe according to the instructions of the kit. After electrophoresis, alkali denaturation, transfer, hybridization, and incubation with a biotin‐labeled mouse anti‐digoxin antibody under the manufacturer’s instruction, the expected positive bands of 4.7 kb are displayed.

### Enzyme‐linked immunosorbent assay (ELISA)

Mouse milk samples were collected as previously reported [13, 29]. The milk samples were diluted three times with PBS and centrifuged at 8000 g for 15 min at 4 °C to obtain whey. Brazzein concentrations in the whey samples were measured by ELISA, as previously described [23]. A polyclonal antibody (1:2000 dilution) raised in rabbits against brazzein standard (CSB‐YP347673PFG, Cusabio, China) and an HRP‐conjugated goat antirabbit IgG (1:10,000 dilution, Cusabio, China) were used as the primary and secondary antibodies,. A brazzein standard curve was established by ELISA using different amounts of brazzein standards dissolved in wildtype mouse whey. The concentrations of brazzein in the whey of transgenic mice were calculated by putting the absorbances at 450 nm into the equation of the standard curve. The ELISA experiment was repeated three times to improve the accuracy.

### Sodium dodecyl sulfate polyacrylamide gel electrophoresis and western blots

After denaturation and electrophoresis in 10% sodium dodecyl sulfate polyacrylamide gel electrophoresis, whey samples were transferred to PVDF membrane (PALL Life Sciences, Port Washington, NY, USA). The membranes were blocked with a no protein blocking solution 1 h at room temperature. A rabbit polyclonal antibody to brazzein standard raised by us (1:2000 dilution) and an horseradish peroxidase (HRP)‐conjugated goat antirabbit IgG (1:10,000 dilution, Cusabio, China) were used as the primary and secondary antibodies. Immobilon Western Chemiluminescent HRP Substrate (WBKLS0100, Millipore, Bedford, MA, USA) was used for chemiluminescence detection. The brazzein standard was used as the positive control.

### Sweet taste evaluations

The transgenic mice milk samples were subjected to a double‐blind sweetness taste as described previously [23]. A total of 18 volunteers participated in the test, including nine males and nine females, aged between 20 and 65 years old, with normal taste acuity. All participants in the sweet taste evaluations were aware of the purpose of the experiment and their roles. Volunteers were asked to rate the sweetness of the milk samples according to the Labeled Magnitude Scale, a semantically labeled scale used to rate the intensity of sensation [30]. All samples were randomly tested three times by all volunteers. The comparison between groups was performed by one‐way analysis of variance (one‐way ANOVA with post‐hoc LSD's test), and *P* < 0.05 was considered statistically significant.

## Results

### pBC1‐brazzein and pBC1‐brazzein‐H31R/E36D/E41A

The synthesized wildtype and triple mutational *des*‐pGlu brazzein genes were cloned into a commercialized pBC1 vector to generate pBC1‐brazzein and pBC1‐brazzein‐H31R/E36D/E41A, respectively. The constructed expression vectors were verified by restriction enzyme digestion and sequencing, which proved that the position and direction of the brazzein cDNA fragments insertions were correct.

### Generation of transgenic mice

In the production of wildtype *d*es‐pGlu brazzein transgenic mice, a total of 50 fertilized eggs were microinjected with the purified pBC1‐brazzein expression vector. After a short period of culture, 43 of them were transplanted into four recipient mothers. In the end, a total of 32 candidate mice were born.

In the production of triple mutational (H31R/E36D/E41A) *des*‐pGlu brazzein transgenic mice, a total of 58 fertilized eggs were microinjected with the purified pBC1‐brazzein‐H31R/E36D/E41A expression vector. After a short period of culture, 58 of them were transplanted into six recipient mothers. In the end, a total of 47 candidate mice were born.

### Detection of exogenous gene insertions in the mice genome

**Fig. 2:**
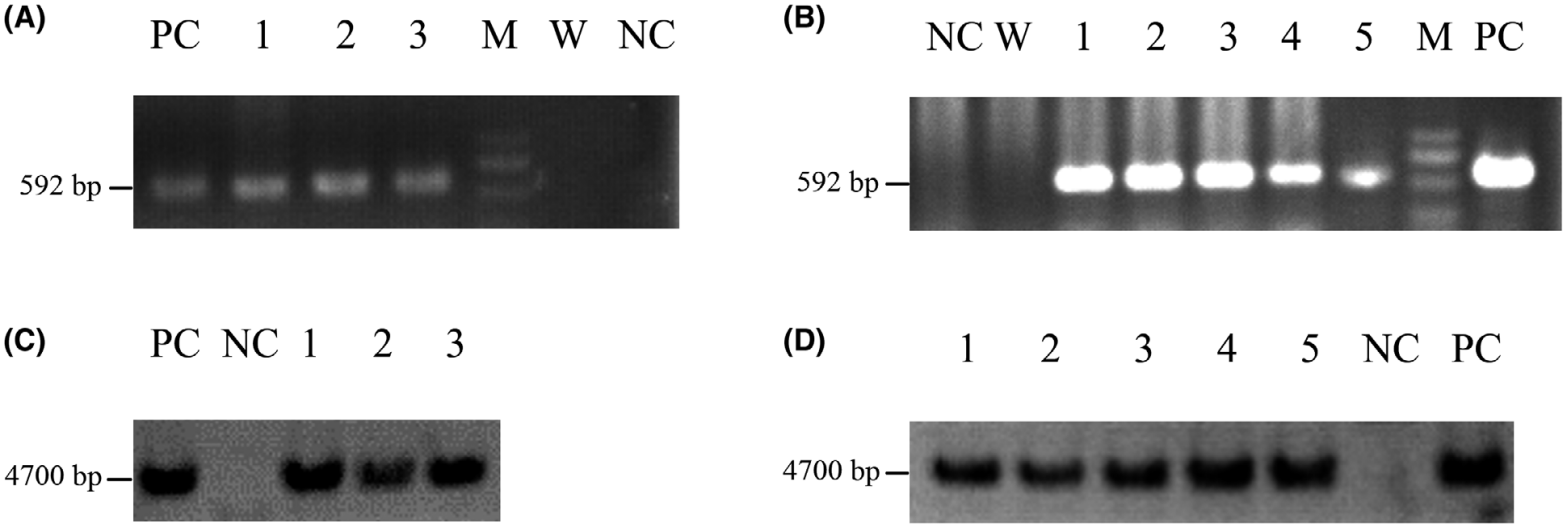
PCR and Southern blot analysis of transgenic mice. (A) PCR analysis of the wildtype *des*‐pGlu‐brazzein transgenic mice. PC: pBC1‐brazzein vector mixed with wildtype mouse genomic DNA; 1–3: Genomic DNA extracted from W1, W2, and W3; M: DL2000 DNA marker; W: water; NC: wildtype mouse genomic DNA. (B) PCR analysis of the triple mutational *des*‐pGlu‐brazzein transgenic mice. NC: wildtype mouse genomic DNA; W: water; 1–5: Genomic DNA extracted from T1, T2, T3, T4, and T5; M: DL2000 DNA marker; PC: pBC1‐brazzein‐ H31R/E36D/E41A vector mixed with wildtype mouse genomic DNA. (C) Southern blot analysis of wildtype *des*‐pGlu‐brazzein transgenic mice. PC: pBC1‐brazzein plasmid; NC: wildtype mouse genomic DNA; 1‐3: genomic DNA of W1, W2, and W3. (D) Southern blot analysis of the triple mutational *des*‐pGlu‐brazzein transgenic mice. 1–5: genomic DNA of T1, T2, T3, T4, and T5; NC: wildtype mouse genomic DNA; PC: pBC1‐brazzein‐ H31R/E36D/E41A plasmid.

Among the 32 wildtype *d*es‐pGlu brazzein transgenic mice candidates, three (female, named W1, W2, and W3, respectively) were proved to be transgenic mice by PCR (Fig. 2A) and Southern blot analysis (Fig. 2C).

Among the 47 triple mutational *des*‐pGlu brazzein transgenic mice candidates, five (T1 and T4 male, T2, T3, and T5 female) were proved to be transgenic mice by PCR (Fig. 2B) and Southern blot analysis (Fig. 2D).

By mating wildtype female mice with the male founder transgenic mice (T1 and T4), we obtained female transgenic offspring for subsequent experiments.

### Determination of the concentration of brazzein in milk

The concentrations of recombinant brazzein in mouse milk were determined by ELISA, as shown in Table 1. The concentration of brazzein is up to 332.59 ± 37.19 mg·L^−1^ in the milk of T3. The female progeny (T1‐F1a and T4‐F1a) also expressed triple mutational brazzein in the milk. These results indicated that not only the founder female transgenic mice, but also the transgenic offspring inherited the characteristics of the parental generation and could express recombinant brazzein in the mammary gland tissue and secrete into milk.

**Table 1:** Brazzein concentrations in transgenic mice milk.

Mouse	W1	W2	W3	T1‐F1a	T2	T3	T4‐F1a	T5
Concentration (mg·L^−1^)	12.38 ± 2.14	146.58 ± 19.76	56.65 ± 7.84	151.85 ± 22.15	26.18 ± 3.77	332.59 ± 37.19	0.63 ± 0.05	29.47 ± 4.31

The results are presented as mean ± SD.

### Western blot detection of brazzein in milk

**Fig. 3:**
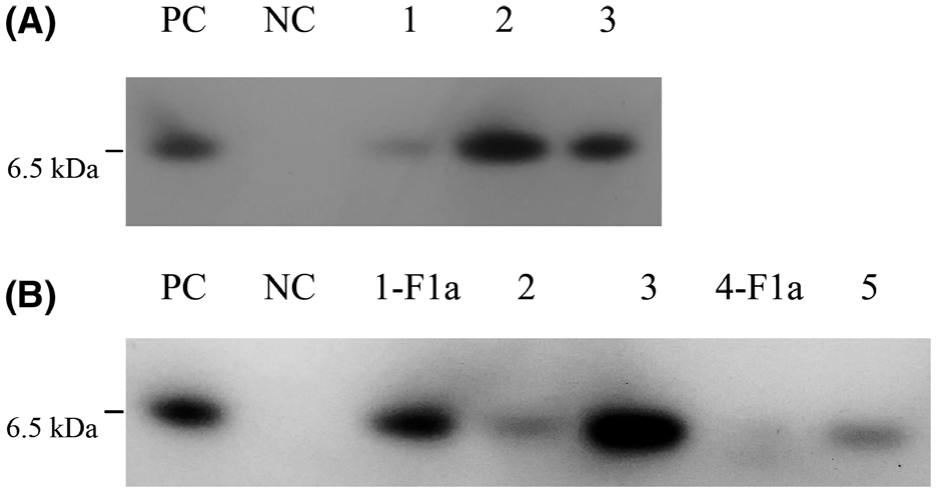
Western blot detection of brazzein in transgenic mice milk. (A) Detection of brazzein in wildtype *des*‐pGlu‐brazzein transgenic mice milk. PC: 2 μg brazzein protein (CSB‐YP347673PFG, Cusabio, China); NC: 10 μL wildtype mouse whey; 1,2, 3: 10 μL whey of W1, W2, and W3. (B) Detection of brazzein in triple mutational *des*‐pGlu‐brazzein transgenic mice milk. PC: 2 μg brazzein protein (CSB‐YP347673PFG, Cusabio, China); NC: 110 μL wildtype mouse whey; 1‐F1a, 4‐F1a: 10 μL whey of T1‐F1a and T4‐F1a; 2, 3, 5: 10 μL whey of T2, T3, and T5.

Corresponding signals were detected in all female transgenic mice milk samples and the positive control, while no signal was detected in wildtype mice milk samples (Fig. 3). T1‐F1a and T4‐F1a were the offspring of founder male transgenic mice T1 and T4, respectively. Both sizes of wildtype and mutational *des*‐pGlu‐brazzein were approximately 6.5 kDa and consistent with the theoretical size.

### Sweet taste properties

The milk of wildtype mice was used as the negative control in the sweet taste evaluation experiment. Milk samples were heated at 100 °C for 10 min to test the thermal stability of brazzein and also for the safety of the volunteers. In order to facilitate the test, all samples were diluted with double‐distilled water after boiling. The milk samples of T1‐F1a, T2, T4‐F1a, and T5 were diluted 16 times, the milk sample of T3 was diluted 32 times, and the milk samples of W1, W2, and W3 were diluted two times as determined by pre‐experiment.

**Fig. 4:**
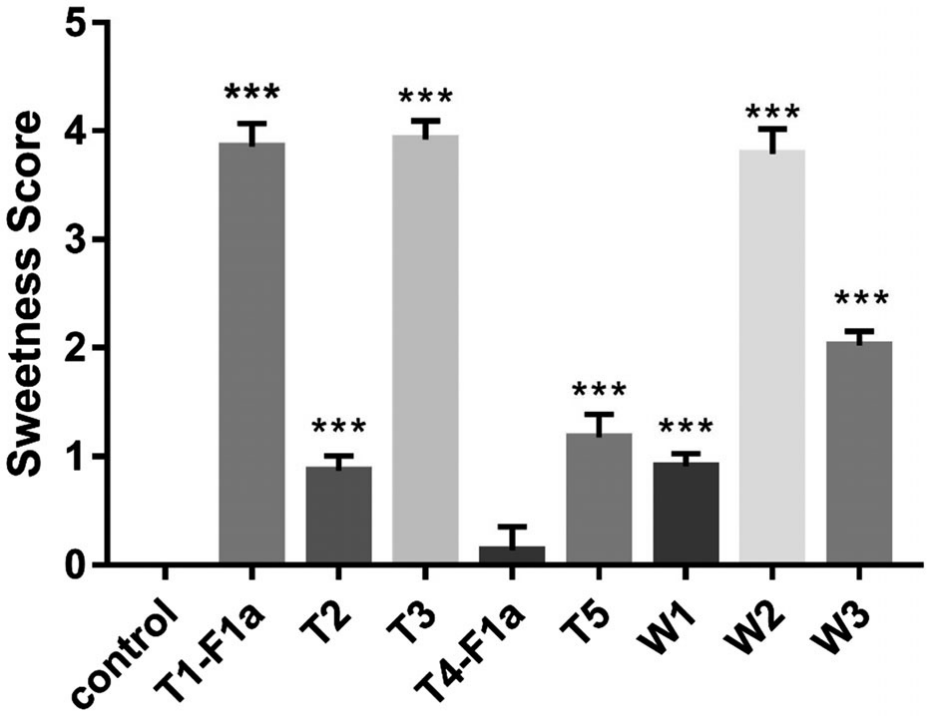
Sweetness test of recombinant brazzein. Control: milk of the wildtype mice; T1‐F1a, T2, T4‐F1a, and T5: 16‐fold diluted milk of the T1‐F1a, T2, T4‐F1a, and T5 triple mutational (H31R/E36D/E41A) *des*‐pGlu brazzein transgenic mice; T3: 32‐fold diluted T3 transgenic mice milk; W1, W2, and W3: 2‐fold diluted wildtype *des*‐pGlu brazzein transgenic mice milk. The results are shown as means ± SD. The comparison between groups was performed by one‐way analysis of variance (One‐Way ANOVA with post‐hoc LSD's test), *n* = 6, ***P < 0.001 compared with control.

The sweetness of 16 times diluted 1‐F1a and 32 times diluted T3 transgenic mice milk was slightly lower than that of the 10% sucrose solution (Fig. 4). The sweetness of 16 times diluted T2 and T5 transgenic mice milk was much lower, similar to a 2% (w/v) sucrose solution. The sweetness of 16 times diluted T4‐F1a transgenic mouse milk was the lowest, and some volunteers felt no sweetness. The result of the sweetness test was positively correlated with the result of the concentration test, which also means the triple mutational *des*‐pGlu brazzein expressed by different lines of transgenic mice had similar sweetness.

The milk of W2, wildtype *des*‐pGlu brazzein transgenic mice, had a sweetness between 6% and 10% sucrose solution after 2‐fold dilution, and its sweetness was about the same as that of 16 times diluted T1‐F1a or 32 times diluted T3 milk.

Duo to some limitations, we were unable to test the unheated brazzein protein. But these results indicated that, on a weight basis, the sweetness of the 10 min incubated triple mutational (H31R/E36D/E41A) *des*‐pGlu brazzein was about 10,000 times that of sucrose, and sweetness of the 10 min incubated wildtype *des*‐pGlu brazzein was about 1200 times that of sucrose. This also means that, after 10 min boiling, the triple mutational (H31R/E36D/E41A) *des*‐pGlu brazzein is about eight times sweeter than the wildtype *des*‐pGlu brazzein.

## Discussion

In the present study we generated wildtype or triple‐mutational (H31R/E36D/E41A) *des*‐pGlu‐brazzein transgenic mice. The results of the ELISA, western blot, and sweetness intensity test indicated that brazzeins expressed in the milk still had sweet tastes even after heating for 10 min. Besides, the triple‐mutational was about eight times sweeter than the wildtype *des*‐pGlu‐brazzein after 10 min of being incubated.

Yan *et al*. reported the expression of *des*‐pGlu brazzein double‐site mutant (D29K/E41K) in the milk of transgenic mice with an expression level up to 4.37 mg·L^−1^. On a weight basis, its sweetness is about 10,000 times that of sucrose after 5 min incubated [13]. In this study, on a weight basis, the sweetness of triple mutant was about 10,000 times that of sucrose even after 10 min being incubated. And we also got much higher expression levels of brazzein in the milk of transgenic mice.

The highest expression level of the triple mutational *des*‐pGlu brazzein in the milk of transgenic mice obtained in this study was 332.59 mg·L^−1^, which is a bit low as a raw material to purify the recombinant brazzein protein. However, the expression level of randomly integrated transgenic animals is related to many factors, such as the integrations of the transgene [31, 32], copy numbers, promoters [33, 34] and the signal peptides [35, 36]. The expression levels of brazzein in the milk of the mice produced in this study were very different, confirming this point. Therefore, we can obtain transgenic animals with higher expression levels by producing more transgenic candidates.

The milk of dairy cows and dairy goats contains various nutrients [37, 38], and is consumed by many people all over the world. In this study, the transgenic mouse milk containing a very low concentration of triple mutational brazzein showed a high level of sweetness even after 10 min being incubated. Therefore, we believe that the mutational brazzein‐sweetened milk can be directly drunk after high‐temperature sterilization. Unlike other medicinal recombinant proteins such as tissue plasminogen activator [39], human C1 inhibitor [40] and monoclonal antibodies [41], there is no need for costly and time‐consuming purification steps. Therefore, the processing cost is greatly reduced, and the development of commercialization is greatly promoted.

When it comes to the isolation of recombinant proteins, we believe that it is not difficult to isolate brazzein from milk. As a sweetener, brazzein does not need to be highly purified because milk‐derived impurities do not affect its use. Due to its sugar‐like taste, high sweetness, high stability, and low calorie [8, 11, 12], we believe it will be a very potential sweetener. But there is a problem; the recognition of genetically modified food in many countries is currently very low. Therefore, both recombinant brazzein and brazzein‐sweetened milk will have considerable resistance in the subsequent commercial promotion.

## Conclusion

The present study has verified that triple mutational (H31R/E36D/E41A) is much sweeter than the wildtype *des*‐pGlu brazzein expressed in transgenic mice milk. Overall, this study provides new insight for producing brazzein or brazzein‐sweetened milk from animals that can be used in food and healthcare applications.

## Conflict of interest

The authors declare no commercial or financial conflicts of interest.

## Data accessibility

The data that support the findings of this study are available on request from the corresponding author. The data are not publicly available due to privacy or ethical restrictions.

## Author contributions

RL, LJ, and YCW conceived the experiments. XML, RL, YCW, and YZ acquired the data. JH contributed materials/analysis tools. RL wrote the article. All authors read and approved the final article.
